# ASMI: An automated, low-cost indenter for soft matter

**DOI:** 10.1016/j.ohx.2024.e00601

**Published:** 2024-10-23

**Authors:** Dylan List, Alan Gardner, Isabella Claure, Joyce Y. Wong, Keith A. Brown

**Affiliations:** a Department of Mechanical Engineering, Boston University, 110 Cummington Mall, Boston, MA 02215, USA; b Department of Biomedical Engineering, Boston University, 44 Cummington Mall, Boston, MA 02215, USA; c Physics Department, Boston University, 590 Commonwealth Avenue, Boston, MA 02215, USA; d Division of Materials Science and Engineering, Boston University, 15 St. Mary’s Street, Boston, MA 02215, USA

**Keywords:** Laboratory Automation, Mechanical Properties, Nanoindentation, Soft Materials, Material Discovery

## Abstract

The automated soft matter indenter (ASMI) is a platform for rapidly performing mechanical characterization of samples with elastic moduli in the range 7 kPa to 67 MPa with a sample acquisition time between 1 and 10 min. It is a low-cost system based upon open-source software, a modified mill, and an educational force sensor with a total bill of materials <$500. This system tests batches of up to 96 samples based on a standard well-plate sample holder without requiring any human intervention. Using the ASMI, users can obtain mechanical data in a programmable manner that enables high-throughput workflows, precisely testing time-dependent phenomena, and integration with other processing steps for closed-loop optimization.

## Graphical abstract

**Figure:**
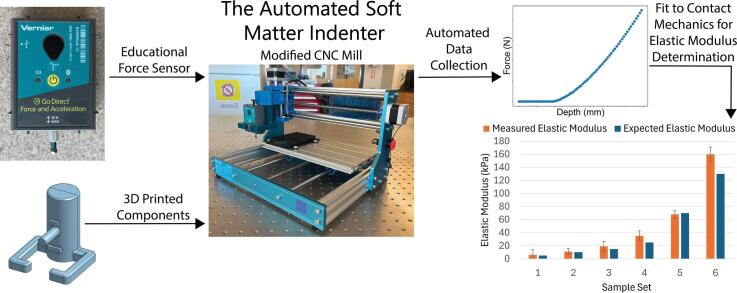

**Specifications table:** 

Hardware name	Automated Soft Matter Indenter (ASMI)
Subject area	• Engineering and materials science
Hardware type	• Measuring physical properties and in-lab sensors
Closest commercial analog	Hysitron TI-950 TriboIndenter
Open source license	MIT License
Cost of hardware	$400
Source file repository	https://doi.org/10.5281/zenodo.13151450

## 1. Hardware in context

Polymers provide an intriguing and practical area for materials development and research due to their wide array of properties and functionalities [1], [2]. Their modular structure and sensitive dependence on processing conditions allows for practically infinite formulations that can be tuned for specific applications ranging from drug delivery to aerospace [3], [4]. While this versatility presents tremendous opportunities, the need to experimentally measure or validate the properties of polymer samples indicates that physical experimentation is a key bottleneck in the materials discovery and development pipeline [5], [6]. This is particularly important when considering properties that depend on both formulation and processing details such as the mechanical properties of hydrogels. To speed up the materials development process, automation allows researchers to delegate tedious, dangerous, or sensitive steps of research and use their time for higher-level thinking or analysis [7]. Indeed, the incorporation of automation in research has accelerated materials development [8], [9], [10]. However, a prerequisite for such acceleration is that effective and versatile hardware is available and that automated solutions be low cost in order to be financially accessible to a wide range of researchers. In addition, open-source solutions allow for researchers to easily share and improve upon each other’s methods. Existing devices such as Jubilee, Sidekick, and LEGOLAS have proved the impact that low-cost, open-source, and easily accessible devices can have for addressing specific needs for material development including tool changing, liquid dispensing, and pH determination [8], [9], [10]. Following the principles of open-source hardware, a device that can mechanically characterize soft and polymeric materials would be a useful aid in the materials discovery process.

Currently, the standard method for determining the mechanical properties of polymers is through the use of mechanical indentation performed by a nanoindenter [11]. This instrument uses an end effector with a precise shape to slightly indent the surface of a material. The resulting force vs. indentation depth profile allows the elastic modulus of the material to be determined using equations from contact mechanics [12]. While these instruments are precise, they are often expensive, have a large setup time, and require substantial user interaction to configure the device and gather the desired data [13]. Previously, we have developed systems for automated mechanical testing that involve the combination of 3D printers, a six-axis robotic arm, and a universal testing machine [14], [15], [16]. A similar system has also been developed that studies the impact mechanics of printed polymer specimens [17]. However, these systems only function with polymers that can be additively manufactured and require gram-scale specimens. Similarly, there are commercially available systems that automatically load dog-bone samples into tensile testing instruments, but these also require macroscopic samples that are not compatible with very soft classes of materials such as gels.

Here, we report the development of the automated soft matter indenter (ASMI), a system for the rapid and low-cost measurement of soft matter mechanical properties. This system relies on the same working principle as a nanoindenter but incorporates a low-cost educational force sensor mounted on a small CNC mill which are both controlled using open-source Python code. Scripts describing typical workflows are provided on github (github.com/dlist26/ASMI). In addition, a detailed flowchart and description of the code is given in the Code Design section. To allow for processing of multiple samples, the system is designed to interface with standard 96-well Society for Biomolecular Screening (SBS) microplates. In this format, quantitative extraction of the material modulus requires compensating for the finite sample size, which we achieve by introducing a model based on finite element analysis. The result is that the system can test a variety of samples with volumes ranging from 100 to 370 µL and elastic moduli between 7 kPa and 67 MPa. A movie showing the operation of the ASMI is provided as Movie S1. Ultimately, this system is easy to assemble and features a bill of materials totaling ∼$400, making it accessible to many researchers. A more quantitative comparison of the ASMI vs. commercial methods for obtaining mechanical property information of soft matter is given in Table 1.

**Table 1:** Comparison between the automated soft matter indenter (ASMI) and commercial systems capable of mechanically characterizing soft matter.

Type	Cost range	Modulus range	Compatibility with automation	Sample format	Comments
ASMI	$400	Gels, elastomers, and other soft matter (7 kPa – 67 MPa)	Automated testing	Well plate or any specimen between 1 and 30 mm tall	Bulk sample properties
Nanoindentation [13]	$10,000 − $100,000	Effectively all materials (low kPa to high GPa)	Automated functions, requires frequent user input	Sheets or films up 5 cm thick	Local sample properties
AFM Nanoindentation [18]	$30,000 − $300,000	Effectively all materials (low kPa to high GPa)	Probe and sample manipulation requires human intervention	Sheets or films up to ∼1 cm thick	Local sample properties
Universal Testing Machine [19]	$10,000 − $100,000	Effectively all materials (low kPa to high GPa)	Automated testing for specific sample formats	Macroscopic dog bones for tensile testing	Bulk sample properties

## 2 Hardware description

When designing the ASMI, the core principles were that the system should be low cost, accessible to those without strong technical backgrounds, and allow for integration into other workflows. These overarching principles led to four key design choices: (1) The hardware foundation of the ASMI is a turnkey CNC mill that allows facile control of the motion of the system in three Cartesian axes. This choice reduces the complexity of the system and the technical requirements of new users considering building a version of the ASMI. (2) The force-sensing element of the ASMI is a low-cost educational tool that is widely accessible, modular, and does not need to be irreversibly modified to be incorporated into the ASMI. (3) The entire system is operated using open-source code written in Python, lowering the barrier to adoption and customization. This choice allows users to start with a baseline testing script, but also customize the code to their own needs by modifying the programs which can be found at github.com/dlist26/ASMI. (4) While the system is in principle able to accommodate any sample thinner than 30 mm on the 26.0 cm × 15.5 cm bed, we have developed hardware and software support for 96-well plates, allowing for the parallelization of characterization and facile integration into other workflows.

**Fig. 1:**
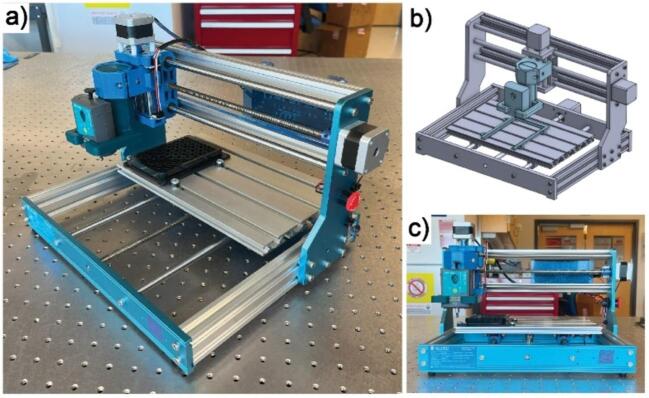
The automated soft matter indenter (ASMI). (a) Isometric view of the system. (b) Isometric view of the 3D model of the ASMI. (c) Front view of the device.

The complete ASMI is comprised of a combination of commercially available or 3D printed components and collectively takes up 0.14 m^2^ of counter space. The assembled device is shown in Fig. 1. Using the provided test script, samples are tested at a rate of, on average, 1 sample per 5 min and up to 96 distinct samples can be tested in one operation without human intervention. The system is also compatible with additional samples and testing protocols including time-dependent testing, creep measurements, and low strain-rate dynamic testing.

### 2.1 Mill choice

The Genmitsu 3018-PROVer Semi Assembled CNC Router was selected as the foundation for the ASMI’s operation for three reasons. (1) This system has a relatively low cost of $269 and comes mostly preassembled, allowing users to quickly start using the device. The equipment which comes with the mill, specifically the three stepper motors, also allows the device to be precisely moved in 10 μm increments, which is important as the step size is a key determinant of indentation measurement precision [20]. (2) The preexisting mounting plate on the mill can easily be adapted to hold a force sensor, which is required for obtaining measurements. Instead of using the router which comes with the mill, a set of compatible 3D printed parts available at github.com/dlist26/ASMI can be securely fastened to attach the force sensor with minimal modification. Additionally, this mill comes with specific safety features that can prevent damage to the system including limit switches on both sides of each axis. Such checks are especially important when developing custom code as these will prevent the machine from unintentionally moving too far. (3) This mill is compatible with a variety of programs for software control. Since it uses a standard GRBL controller, many libraries already exist which can be used with the device, allowing users to intuitively understand and customize how the device operates.

### 2.2 Sensor choice

**Fig. 2:**
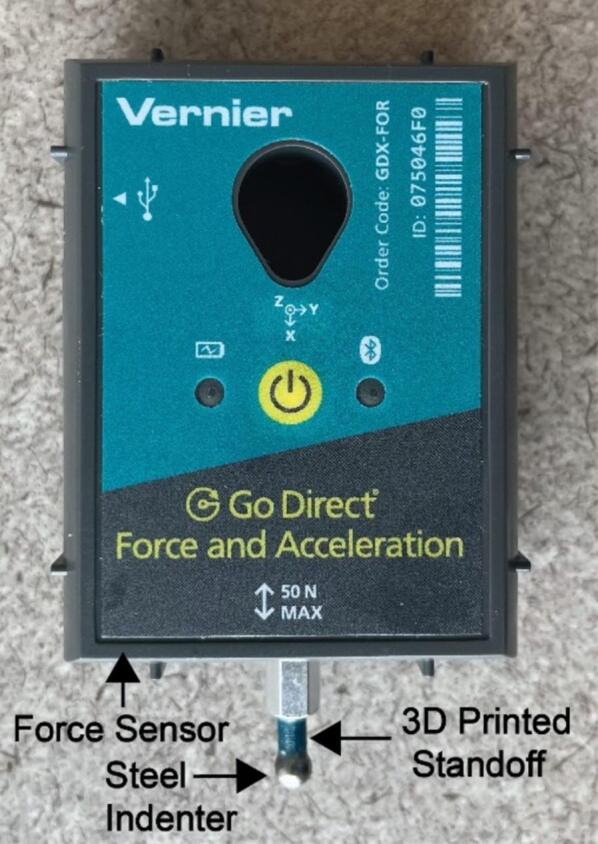
The force sensor with the attached indenter.

In an effort to design the system with an emphasis on accessibility, the Vernier Go Direct® Force and Acceleration Sensor was chosen to measure the indenting force F because of its precision and its compatibility with open-source software. This sensor can communicate over USB or Bluetooth and has a simple Python programming interface for controlling and programmatically reading data for analysis in real time. In addition, the ±50 N range of this sensor allows samples with a wide set of estimated elastic moduli to be tested [21]. Lastly it allows for an end effector to be easily attached without causing any permanent damage to the sensor. This is important because an end effector with known shape and mechanical properties is required to calculate the elastic modulus of a sample using the equations of Hertzian contact mechanics. Fig. 2 shows the force sensor assembly including the custom attached indenter.

**Fig. 3:**
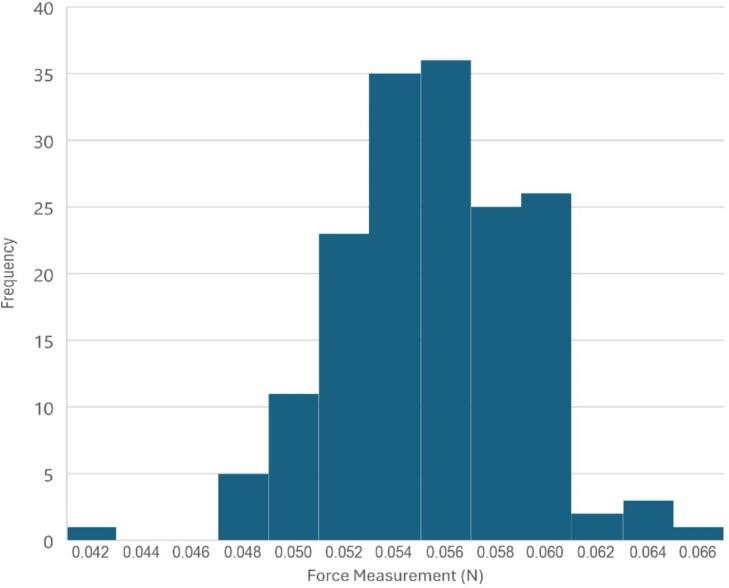
A histogram of data collected by the force sensor while freely suspended in air.

To begin quantifying the sensor and its operating range, it is necessary to determine the precision and accuracy of the sensor. Initially, we estimated the precision of the sensor by taking repeated measurements while the probe was not in contact with a sample, which is a direct measure of the stability of the device. We performed a series of 168 measurements which are shown in Fig. 3. These measurements were found to vary with a standard deviation of 0.0038 N, representing a relatively small source of error relative to the force range.

When developing the strategy for materials characterization, a priority was fidelity with standard contact mechanics models to facilitate analysis. In particular, the ASMI end effector was designed to feature a spherical tip. Considering the sample to be an elastic half-space, Hertzian contact mechanics can be used to estimate the ideal force $F_{\mathrm{Hertz}}$ vs. indentation depth d, and indenter radius R as [22]

(1) $$F_{\mathrm{Hertz}}=\frac{4}{3}E^{*}R^{0.5}d^{1.5}$$

where $E^{*}$ is the reduced modulus of the system given by,

(2) $$E^{*}={\left(\frac{1-\nu_{s}^{2}}{E_{s}}+\frac{1-\nu_{i}^{2}}{E_{i}}\right)}^{-1}$$

where $\nu_{i}$ and $E_{i}$ are the Poisson’s ratio and elastic modulus of the indenter while $\nu_{s}$ and $E_{s}$ are the Poisson’s ratio and elastic modulus of the sample. As the metal indenter with $E_{i}=200$ GPa is at minimum 100-fold stiffer than the samples under consideration, its contribution may be neglected.

The combination of the quantification of the force sensor, the geometry of the indenter, and the contact mechanics equations allows us to estimate the range in $E_{s}$ over which the system is expected to generate accurate results. In particular, a material is deemed to be too soft to test if F does not exceed twice the noise threshold when d= 0.5 mm. Here, the noise threshold is estimated as twice the standard deviation of the free-space value (*i*.*e*. 8 mN). This criterion was chosen as the device interprets a force measurement greater than this threshold as an indication that the probe has come into contact with the sample. This 0.5 mm depth was chosen so that the device could still collect sufficient data to perform calculations even if it did not correctly determine the height at which the indenter made first contact with the sample. This analysis suggests that suitable samples must feature $E_{s}\geq$ 7 kPa. At the other limit, a sample is deemed to be too stiff to accurately test if F> 50 N when d= 0.5 mm. This criterion ensures that enough data can be collected to perform calculations without exceeding the sensor’s limit of 50 N. Using these constraints, the stiffest sample that could be tested with the ASMI would $E_{S}\leq$ 67 MPa.

### 2.3 Code design

**Fig. 4:**
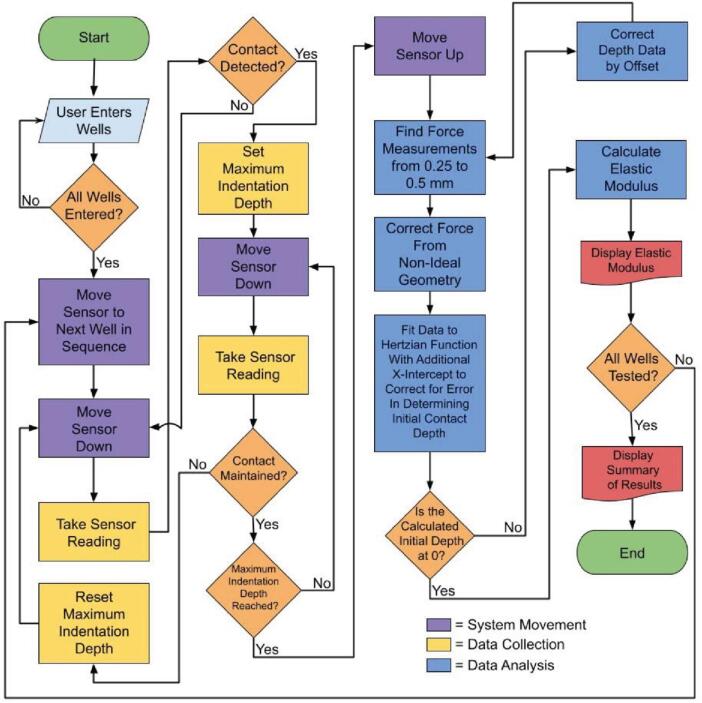
A flow chart overview of the “measure” script used to obtain elastic modulus measurements.

The code that controls the ASMI was written in Python and combines several open-source libraries to ensure that users can understand and modify the code to fit their needs. All the code can be found at github.com/dlist26/ASMI, including additional scripts to test and troubleshoot the device. The main “measure” script can be broken down into three interdependent processes: moving the sensor, taking measurements, and analyzing the data. Fig. 4 provides an overview of this program.

To move the sensor, code from the python_to_GRBL GitHub repository was used which sends movement commands to the device and waits for it to reach its end state before sending the next movement. This is done through the use of g-code, a programming language that runs the CNC mill. At a basic level, g-code is a scripting language that provides line-by-line instructions on how to move the various motors in the system [23]. Then, when desired, measurements from the sensor are collected using Vernier’s Go Direct® library and its associated functions. The use of these libraries allows for users to easily customize the device to their own needs using preexisting functions and code that has already been developed.

As a proof-of-concept use case, we developed a software loop that allows arbitrary wells in a 96-well plate to be measured using the ASMI (Fig. 4). This configuration was chosen because the use of such well plates is already common, which allows the system to interface with standard laboratory equipment and protocols. In addition, this setup allows for a variety of sample sizes, giving the user flexibility for what the system can test. Lastly, the choice of these well plates in combination with the indenter is useful in determining bulk properties, in contrast to nanoindentation, which can be restricted to measuring the properties of the top layers of a sample that potentially have different surface characteristics due to the small range and fine geometry of nanoindentation. By using macroscopic samples in connection with a large indenter and sensor that can measure larger forces, properties of an entire sample can be measured.

The custom-developed measurement software begins with prompting the user to enter which wells in the well plate they would like to test, giving them the ability to input the desired wells one at a time, as entire rows, as entire columns, or to indicate that the entire plate should be tested. The user will finally be asked to enter the approximate Poisson’s ratio of the samples with the option to enter the same ratio for every sample or to enter each sample’s ratio individually. Once programmed, the system proceeds to test each well one at a time, requiring no further user intervention. The built-in quick setup is useful for ensuring that the system is accessible.

For each well that is tested, g-code is generated and sent to the mill to move it to the x-y position of the well so testing can begin. Once the machine has moved above the target well, the sensor takes a set of 10 readings whose average is used as a baseline force which can be used throughout the test as a reference. Then, g-code is sent to the mill which moves it downwards in steps of 20 μm. After each movement, F is measured to determine if contact has been made with the sample. The sensor is said to be in contact with the sample when F is greater than two standard deviations away from the average force measured in free space before the current well was tested. Due to fluctuations, it is possible that the sensor falsely indicates that contact has been made. To safeguard against this, the code is designed to look for continuous contact as determined by increasing F at each increasing depth. Since each sample only needs to be indented by 1 mm to ensure proper measurements, determining where contact was first made is important for estimating how far the sample was indented at each corresponding measurement. To do this, each time the code determines the sensor has made contact after the previous reading had indicated no contact, the code sets a minimum height to which the machine will need to move while indenting the sample. If a future reading falls back below the threshold, the code interprets the previous reading or readings as a false alarm, and this minimum height is reset.

**Fig. 5:**
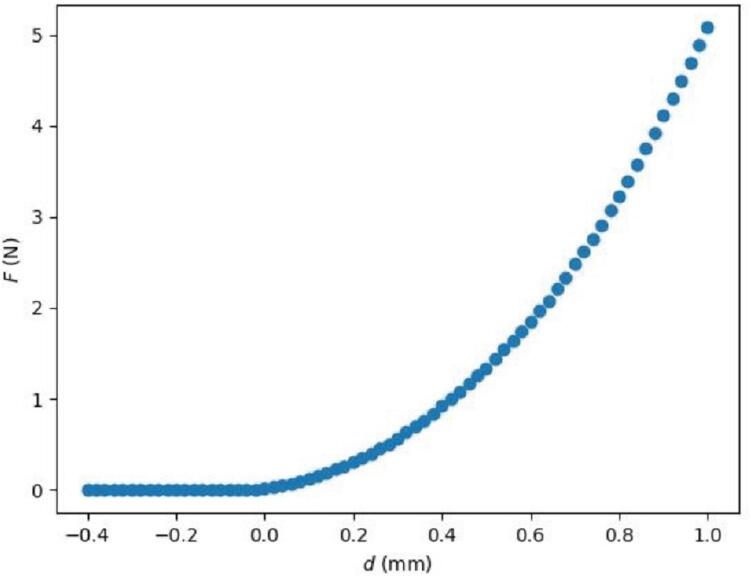
Typical relationship between measured force F and indentation depth d for a measurement of a polydimethylsiloxane (PDMS) sample in a well of a 96-well plate.

As the probe continues to indent the sample, the sensor will eventually make continuous readings above the force threshold due to contact with the sample. The system will continue to indent the sample until it reaches d= 1 mm relative to the first measurement showing contact from the string of continuous contact measurements. Indenting this far ensures that the sample is being elastically deformed for proper measurements while enough data is being collected to accurately determine $E_{s}$ even if the initial contact height was not precisely determined. Additional error prevention is also implemented in this code to prevent damaging the device or its components. Specifically, if the sample is too stiff and F rises to levels that may exceed the limits of the sensor, or if the probe has moved so far that it may hit the bottom of the plate, the system will automatically stop lowering the sensor and analyze the data that was collected. Measurements that have been completed are saved to files during the process so that, in the event that something causes the system to stop running in the middle of a series of tests, the data that was collected can be analyzed. Fig. 5 shows a typical F vs. d profile collected on a polydimethylsiloxane (PDMS) sample in a 96-well plate.

The final part of the measurement code analyzes the data that was collected. For each well, F corresponding to the indentation experiment of the sample is collected and zeroed based on the average baseline force measured before indentation. While Eq. (1), which represents Hertzian contact mechanics, is useful for determining $E_{s}$ from this data, its direct application necessitates that the sample is an infinite half-space. Deviations from this, such as the sample having finite thickness, can cause corrections to this equation to become necessary similarly to how we have previously corrected this equation in the case of thin films using finite element analysis [24], [25]. Since the samples in consideration here are confined in wells with known geometry, F measured by the sensor exceeds what the Hertzian equation predicts, making the sample appear stiffer.

**Fig. 6:**
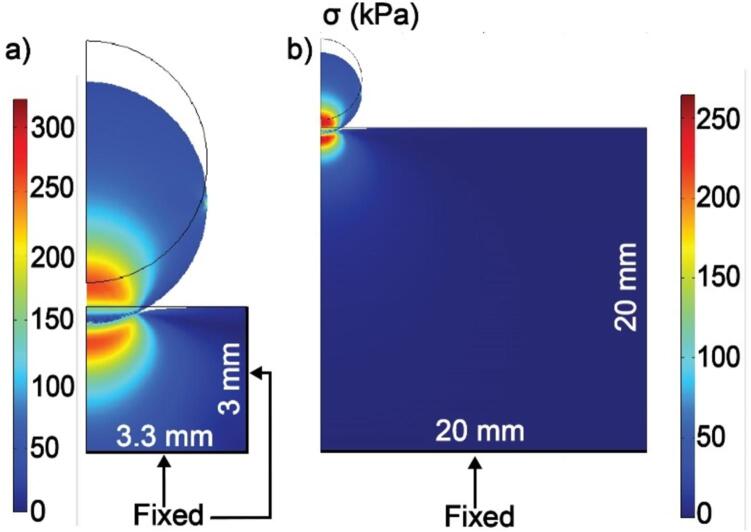
COMSOL Multiphysics 2D axisymmetric finite element calculations of von Mises stress σ in geometries relevant to the ASMI. (a). A cylindrical sample with radius 3.3 mm and height h= 3 mm is chosen to model a 96-well plate. (b). A sample with radius 20 mm and h= 20 mm to approximate an elastic half-space. All boundaries not noted as fixed are free.

**Fig. 7:**
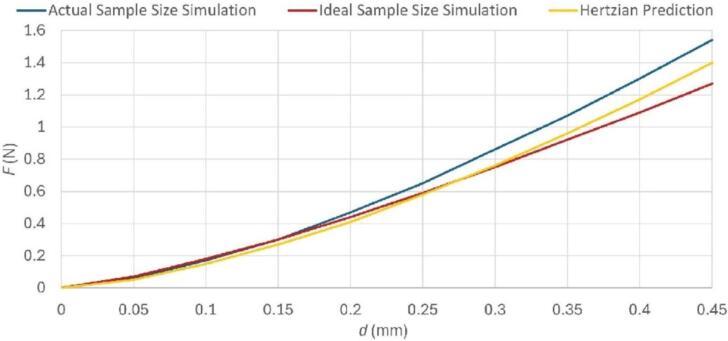
Comparison of simulated force $F_{s}$ and ideal force $F_{\mathrm{Hertz}}$ corresponding to a sample with modulus $E_{s}=2$ MPa, height of 3 mm, and Poisson’s ratio $\nu_{s}=$ 0.3 that is indented by a rigid indenter with a 2.5 mm radius. The blue curve corresponds to the geometry depicted in Fig. 6a, which represents the well plate tested by the ASMI. The red curve corresponds to the geometry depicted in Fig. 6b, which is intended to approximate an infinite half-space. The yellow curve shows $F_{\mathrm{Hertz}}$ computed using Eq. (1).

To account for the apparent stiffening of the sample due to the confinement in the well, we compute a correction for F that was determined using finite element analysis (FEA) calculations performed in COMSOL Multiphysics. Specifically, we constructed a 2D axisymmetric system featuring a spherical indenter with a 2.5 mm radius and a cylindrical sample, as shown in Fig. 6. To validate that this process was correct, we first computed the mechanical response of a large region designed to approximate an elastic half-space that was 20 mm in depth and 20 mm wide. These results were compared to calculations of a sample designed to mimic a single well in a 96-well plate that was 3.3 mm wide and had heights h ranging from 3 to 10 mm. We specified $E_{s}=2$ MPa, although since this was much smaller than $E_{i}$, its specific value does not affect the measured correction factor. In contrast, $\nu_{s}$ is not generally known *a priori* and is expected to affect the correction factor. Nevertheless, it is expected to be in consistent ranges for different classes of materials with $\nu_{s}\approx 0.3$ for glassy polymers and $\nu_{s}\approx 0.5$ for gels. Thus, we computed cases in which $\nu_{s}=$ 0.3, 0.35, 0.4, 0.45, and 0.49. In each calculation, we compute a correction factor $\phi \left(h,d,\nu \right)$ which is the ratio between the computed force $F_{s}$ and $F_{\mathrm{Hertz}}\left(d,\nu \right)$ as given in Eq. (1). Prior to analysis, the user must select $\nu_{s}$ and h is determined using the measured contact point. Fig. 6 shows a comparison between σ, the computed von Mises stress from FEA, for both a 3 mm tall sample and the sample representing an ideal elastic half-space, both with $E_{s}=2$ MPa and $\nu_{s}=$ 0.3. As a typical result, Fig. 7 shows the simulated results for this combination of *h* and ν.

**Fig. 8:**
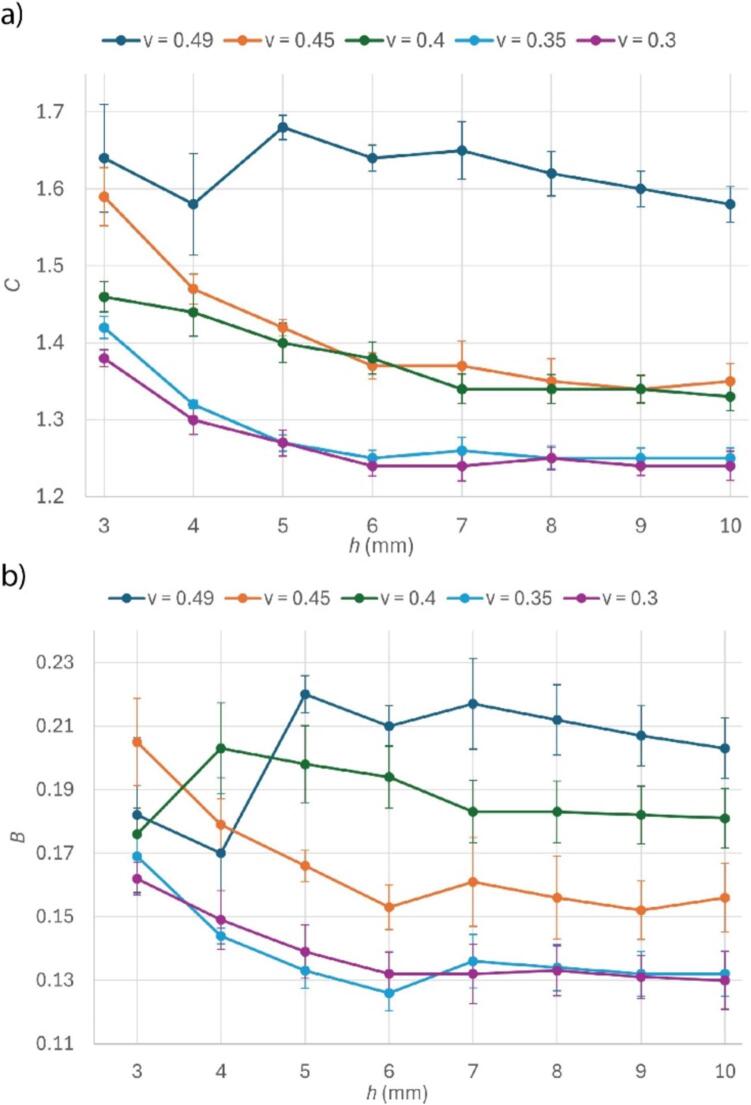
Correction factor equation coefficients computed for each h and $\nu_{s}$ combination. (a) Computed values of multiplicative parameter *C*. (b) Computed values of exponential parameter *B*. Error bars denote confidence intervals from fitting.

Each of the 40 correction equations for every configuration of Poisson’s ratio and sample height take the empirical form

(3) $$\phi \left(h,d,\nu \right)=C\cdot d^{B}$$

where *C* and *B* represent constants from the power fit equations determined for each set of simulations. The values of *C* and *B* for different values of *h* and ν are shown in Fig. 8.

**Fig. 9:**
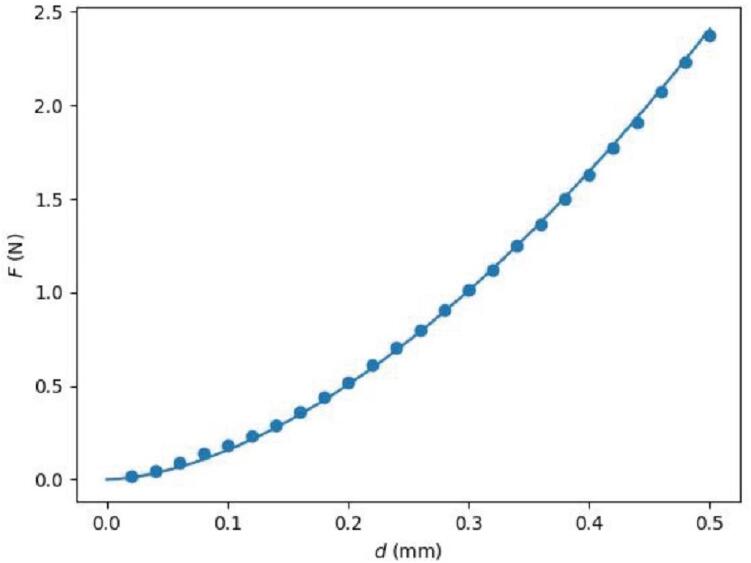
Experimental F vs. d along with a fit line given by Eq. (4).

**Fig. 10:**
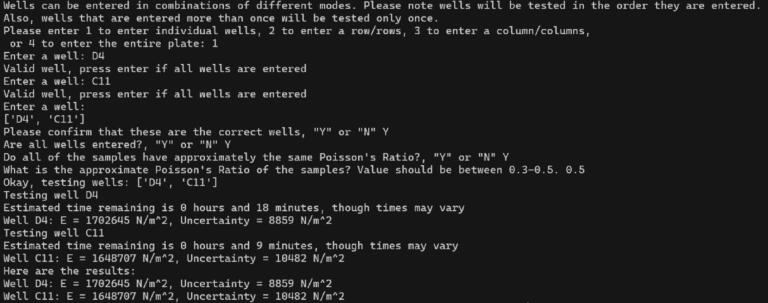
Example output corresponding to one measurement by the ASMI.

While the computation can produce ϕ for any combination of ν, h, and d, this cannot be directly applied to correct F because it is crucial that d=0 indicates the point of first contact in both the simulation and experiment. Even with the contact detection script, the contact point may need to be shifted due to the discrete nature of the motion of the system. This necessitates an iterative process where fitting is used to estimate a contact shift $d_{0}$. Specifically, F corresponding 0.25 ≥d≥ 0.5 mm is fit to

(4) $$F=\frac{4}{3}\phi \left(d-d_{0},\nu ,h\right)\frac{E_{s}}{1-\nu_{s}^{2}}R^{0.5}{\left(d-d_{0}\right)}^{1.5},$$

to estimate $E_{s}$ and $d_{0}$. This range in d was chosen so that d is large enough that the indenter is definitively in contact with the sample but still shallow relative to the geometry of the system to maximize the degree to which the geometric constraints of the Hertzian model are valid. After fitting, if $d_{0}$ is larger than 0.01 mm, this value will be subtracted from d and the fitting process is repeated. Iteratively performing this process ensures that a consistent range of data is selected and that the corresponding factor is correctly applied. An example final fit is shown in Fig. 9. The final value for $E_{s}$ along with uncertainty from fitting is reported to the user, as shown in Fig. 10.

## 3 Design files summary

**Table:** 

**Design file name**	**File type**	**Open source license**	**Location of the file**
Force Sensor Mount	STL	MIT License	https://github.com/dlist26/ASMI/blob/main/ASMI_ForceSensorMount.zip
Plate Holder	STL	MIT License	https://github.com/dlist26/ASMI/blob/main/ASMI_WellPlateHolder.zip
Standoff	STL	MIT License	https://github.com/dlist26/ASMI/blob/main/ASMI_Standoff.zip

**Force Sensor Mount**: A set of two 3D printed components which attach the force sensor to the CNC device.

**Plate Holder**: A 3D printed component to hold a standard 96 well SBS microplate.

**Standoff**: A 3D printed component to increase the maximum depth the indenter can reach.

**Fig. 11:**
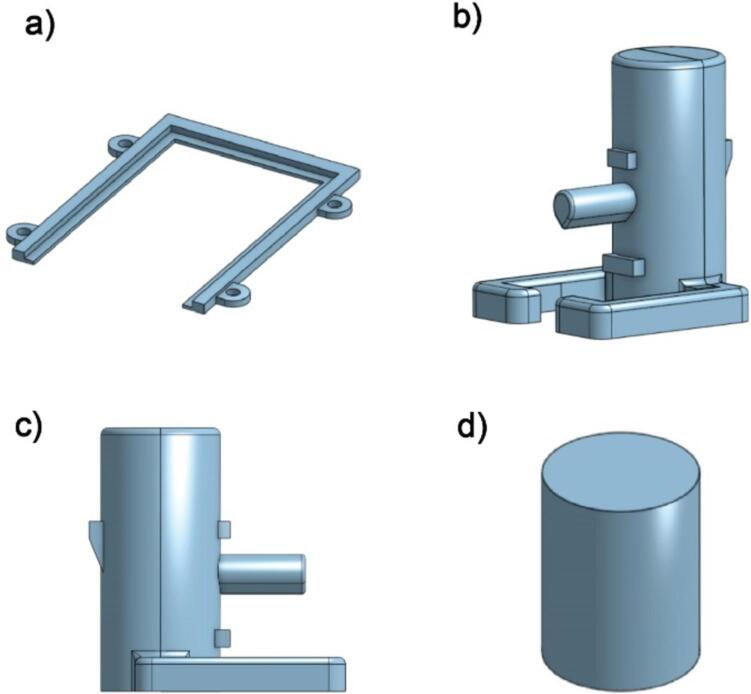
3D models of all 3D printed components in the ASMI. (a) Holder for the well plate. (b) Isometric view of force sensor mounting pieces. (c) Side view of force sensor mounting pieces. (d) Indenter standoff.

The models for all the 3D printed parts are shown in Fig. 11.

## 4 Bill of materials summary

**Table:** 

**Designator**	**Component**	**Number**	**Cost per unit −currency**	**Total cost −** **currency**	**Source of materials**	**Material type**
Gantry	3018- PROVerSemi Assembled CNC Router Kit	1	$269.00	$269.00	Product Link	Metal
Force sensor	Vernier Go Direct® Force and Acceleration Sensor	1	$119.00	$119.00	Product Link	Polymer
Spherical indenter	Steel ball bearing	1	$9.99	$9.99	Product Link, Part 929K39	Metal
Mounting components	Set of 4T-Slotted fasteners	1	$7.11	$7.11	Product Link, Part 4976N22	Metal
Epoxy	Epoxy	1	$7.00	$7.00	Product Link	Polymer

## 5 Build instructions

Step 1: Print all 3D printed components: the force sensor mounting pieces, the plate holder, and the standoff. Any rigid plastic would be acceptable. In our realization, these are produced using polylactic acid (PLA) using fused filament fabrication (FFF).

Step 2: Assemble the gantry following the included instruction manual. All instructions about the spindle can be ignored and this component should not be attached to the system.

**Fig. 12:**
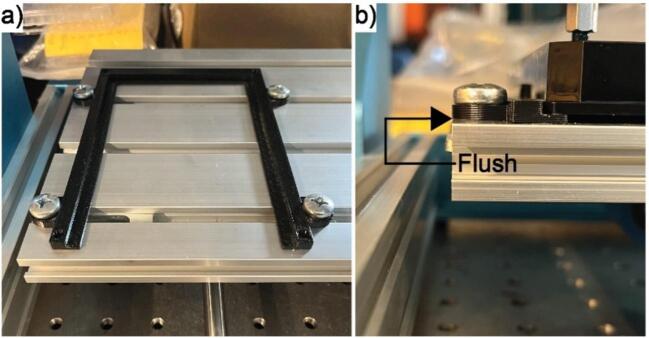
Most convenient placement of the well plate holder. (a). The open side of the well plate holder should be facing forward. (b) The outermost left side of the well plate holder should be flush with the left side of the metal floor of the gantry.

Step 3: Attach the plate holder to the metal floor of the gantry using the T-Track mounting pieces. It is easiest to align the holder such that the open side is facing forward and the outside edge of the circular flanges on the left are flush with the side of the metal floor as shown in Fig. 12.

Once the left side is fastened to the metal floor, place a standard 96-well SBS microplate into the holder. Push the right side of the holder so that the plate is firmly held in place, and then fasten the right side of the holder to the floor of the gantry using the T-Track mounting pieces. Ensuring that the holder is properly aligned is very important to gathering data and avoiding errors associated with the indenter colliding with the well plate.

Step 4: Assemble and install the force sensor. Begin by connecting the steel indenter to the standoff using epoxy, ensuring that the indenter is as centered on the standoff as possible. Gloves are recommended when handling epoxy. Once cured, use the epoxy to connect the other end of the standoff to the sensor, again ensuring that it is vertically aligned and centered. The standoff should also fit in the screw groove of the sensor to aid with this. By using the standoff, the indenter can reach farther down into wells, allowing shorter samples to be tested. The completed sensor-indenter assembly should look like Fig. 2.

**Fig. 13:**
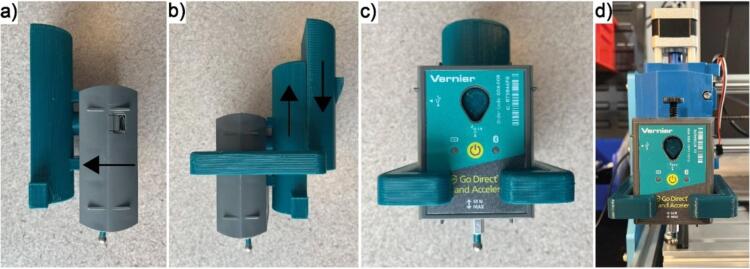
Assembly guide for mounting the force sensor to the CNC. (a) Slide the sensor onto the prong on the front part of the mount. (b) Slide the back part of the mount over the front part and sensor. (c) The fully connected sensor and mount. (d) Slide the top part of the mounting pieces into the spindle holder on the CNC and tighten the screw to securely hold everything in place.

Once all the epoxy has cured, the force sensor can be loaded into the force sensor mounts and this subassembly can be attached to the rest of the CNC mill following the steps shown in Fig. 13.

Step 5: Connect the mill and sensor to a computer, each via USB. This system has been verified to run on both Windows 10 systems and Raspberry Pi running Linux.

Step 6: Initialize the device using Candle, a free CNC software program that allows the device to be configured. Candle can be used on both Windows and MacOS. The initialization with Candle can be done on a separate device if the permanent device that the system will be connected to cannot run this software.

**Fig. 14:**
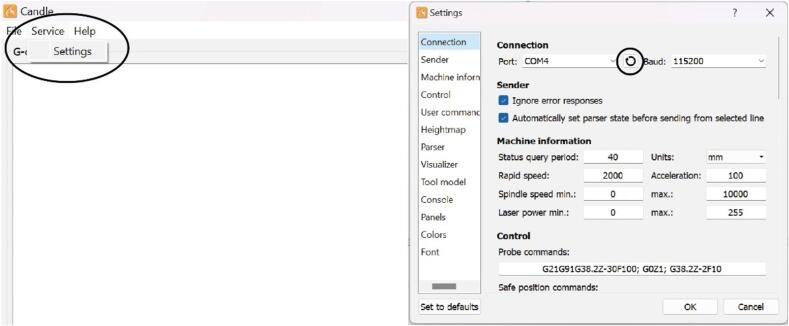
Correct way to find the port to which the device is connected.

**Fig. 15:**
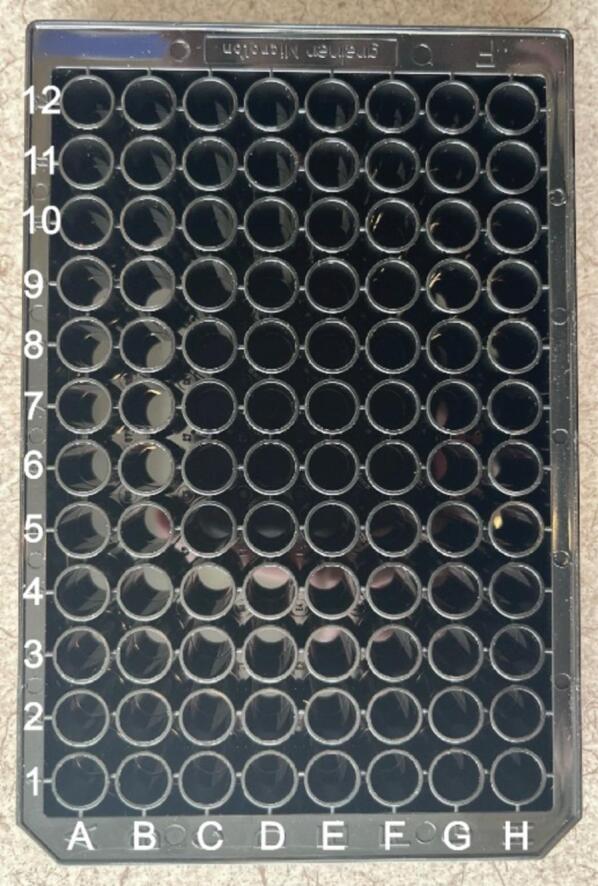
The well numbering of a well plate used by the ASMI**.**

Fig. 14 shows the steps needed to initialize the gantry using Candle. First, click the service tab in the top left and press the refresh button next to the port on the window that pops up. This will display the name of the port which the device is connected to which will be useful when configuring the code. Place a stock well plate into the system for the purposes of alignment. Using the manual positioning capabilities of Candle, move the device in the x and y directions using the buttons on the right panel of the software so that the indenter sits exactly above the well in the bottom left corner, referred to as well A1. A full diagram of the well names that the device uses is shown in Fig. 15.

Record these x and y values as these will later be used to configure the Python code. Additionally, confirm that the distance between wells is also set up correctly by moving the device to multiple other wells and computing their separation in x or y. In a standard well plate, the distance between the centers of neighboring wells should be 9 mm.

**Fig. 16:**
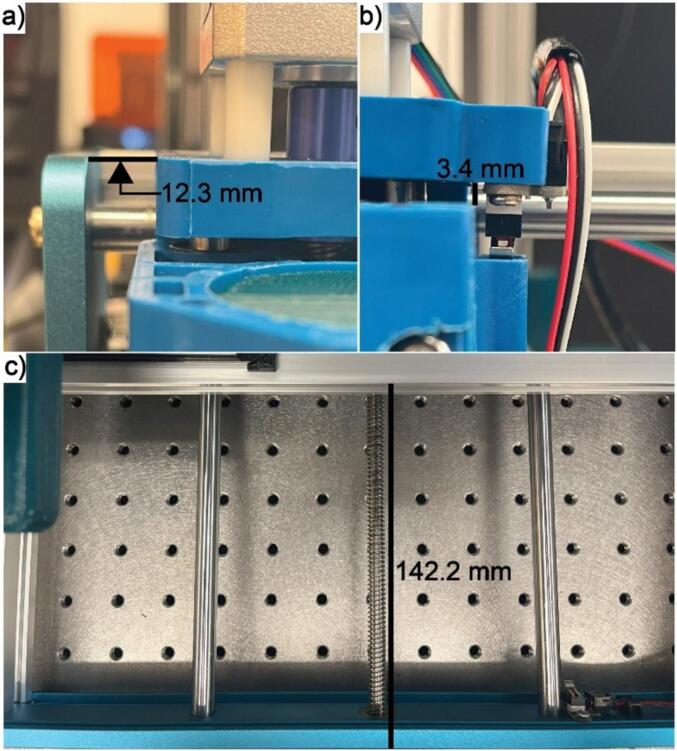
Home position of the ASMI. (a) Correct location for the home x position. (b) Correct location for the home z position. (c) Correct location for the home y position.

Step 7: Rehome the gantry by pressing the home button on the top part of the right-side panel of the Candle software to return the device to its home coordinates. When at its home position, the blue plastic gantry which moves in the x-direction should be 12.3 mm to the right of the metal blue wall, the blue mount which moves in the z-direction should be 3.4 mm below the plastic blue gantry, and the front face of the metal tray should be 142.2 mm from the front of the bottom blue plate as shown in Fig. 16.

Step 8: Configure the code. Download the ASMI repository found at github.com/dlist26/ASMI onto the device that will permanently be connected to the system. The code is known to work in Python 3.8 and requires the following libraries to be installed: serial, godirect, numpy, matplotlib, and scipy. Enter in the initial values and offsets into the x_init, y_init, and offset variables in the “move”, “measure”, and “measure_over_time” programs. Additionally, set the GRBL_port_path variable to the corresponding port (e.g. “COM4”) the CNC device is plugged into in those same programs as well as the “button”, “home”, and “custom_measure” scripts. Lastly, set the file path variable path to be the folder where the code files and measurement data will be stored in the “measure”, “measure_over_time”, “analysis”. “measure_over_time_analysis”, and “custom_measure” programs. Users may also adapt the code to fit their needs.

Step 9: Ensure the device is working properly by running the provided test scripts. Start by running the “move” script to check that the device moves properly. This script allows users to enter wells and the system will move to the x-y position of each of these wells before returning to its home position. If this completes successfully, use the “button” script to check that the force sensor is working and that the program can communicate with the CNC device. This script will move the force sensor back and forth if it measures a force with a magnitude greater than 0.1 N. If this works as expected, the system is ready to be tested.

Step 10: Run the “measure” script to gather data on a sample with known properties to test that the system is obtaining correct results. Once valid measurements have been confirmed, the system is ready to use.

## 6 Operation instructions

The instructions in this section will detail how to use the ASMI to measure samples in a 96-well plate, but users can configure it to run in any way they would like as well as make it compatible with different types of sample holders.

Step 1: Load the samples into the system. The provided version has been developed to accommodate samples in a standard 96 well SBS microplate. Place the samples into the holder by pushing the well plate as far back as possible.

Step 2: Make sure the sensor is in its home position as indicated by Fig. 16. If this is not the case, run the “rehome” script. If the system does not move or moves to an unexpected location, the device needs to be rehomed using Candle and the “reset_home” script needs to be run.

Step 3: Check the alignment by running the “move” script and checking that the sensor correctly aligns itself with a few wells. If this is not the case, use the “custom_measure” program to specify the location and spacing between the wells when testing. This script begins with having the user jog the sensor to the x-y position of wells A1, H1, and H12, and uses these coordinates to determine the proper location and spacing of the wells.

Step 4: Make measurements and collect data. If everything is aligned properly, run the “measure” program and enter in the wells to be measured following the prompts from the script. If multiple measurements need to be made over a set period of time for particular samples, use the “measure_over_time” program. Any program which takes measurements will ask for a file name to save the data to. The program will start testing wells and will provide an estimate of how much time remains in the testing process. This timing is dependent upon the height of the samples, with taller samples requiring less time to test. The machine also requires samples to be a minimum of 3 mm tall to be able to collect accurate data. If the samples are shorter, the system will display that either the sample was too short or too soft to test since the sensor could not detect contact reliably. While the machine runs, the user is free to work on other tasks and return when the testing has finished.

Step 5: Obtain results. Once all wells have been tested, a summary of the results will be displayed. In order to get the results for a specific well, run the “analysis” script for tests done using the “measure” and “custom_measure” programs, and the “measure_over_time_analysis” script for measurements made using the “measure_over_time” program. Either of these analysis scripts will ask users to input the name of the file the test data was saved to, and enter in the well they would like the results for.

Below are some common issues and how to troubleshoot them:

• Running the “measure”, “custom_measure”, “measure_over_time”, or “button” script results in “No Bluetooth Adapters Found” or no force sensor found:
  The force sensor is not properly plugged in. Unplug it, reconnect it, and try again. Check that the correct port is being identified in the code. To properly test this, use the “button” program to check that the sensor is properly taking measurements.
• Running the “measure” script results in “[Errno 2] could not open port …”:
  The code for the force sensor was not properly closed during the last tests, likely due to the tests being interrupted. Check that the ASMI and the computer it is connected to is plugged in and properly powered. Also, make sure the ASMI is connected to the correct USB port on the computer as indicated by the USB port entered in the code files. Then, rerun any measurement taking script.
• The device doesn’t move after inputting wells in the “move”, “measure”, “measure_over_time”, or “custom_measure” programs, or after applying force to sensor in the “button” program:
  Turn the CNC off using the switch on the back, unplug everything, plug it all back in, and turn the machine on again. Also check that the CNC is plugged into the proper port on the computer and that the emergency stop button on the right side of the CNC has not been accidentally pushed. To test that the CNC works properly, run the “move” script to move the sensor to a few locations to make sure it is running properly. Then, restart the tests.
• The device stays still for a while:
  This issue typically resolves itself within a minute if it occurs. If the device stays still for longer than that, press Control + C and follow the directions below for if the CNC is frozen when returning to it.
• The CNC is frozen when returning to it with no error on the screen:
  Something might have happened during the test which caused either the computer or the device to lose power. Press Control + C. Run the “home” program and check that the device has returned to its proper home location. If it has not, the device needs to be rehomed following the rehoming procedure below. Gather any data that was collected using the “analysis” program, and then test the wells that weren’t tested before using the “measure” program.
• The system needs to be stopped in the middle of a test:
  Wait for the device to stay still for a moment and press Control + C. Run the “home” script to move the device back to its home position.
• The machine needs to be rehomed:
  Either open the Candle software on the connected computer or open it on an alternate computer. If it needs to be downloaded, follow the directions at: https://docs.sainsmart.com/article/7c20d7zaw3-how-to-install-candle-grblcontrol-for-windows. If the device is being rehomed on a temporary computer, the USB-B port on the back of the mill will need to be reconnected to the permanent device after rehoming the machine. Make sure that Candle is accessing the correct port as shown in Fig. 14 and press the home button. Once homed, reconnect the device to the original computer and run the “reset_home” program.
• Using the “home” program doesn’t return the device to its proper home position or causes an error:
  Follow the rehoming directions above.
• The indenter keeps crashing into a wall of the well plate:
  The well plate being used does not follow the same dimensions as a standard 96 well SBS microplate, however, if the well plate still has uniform distances between the wells, it can be tested. Use the “custom_measure” program which allows locations of wells and the distances between them to be specified. Follow the directions on screen to move the indenter to 3 wells, and using the coordinates from those wells, the device can determine where each well is located. The more accurate the placement of the indenter above the original 3 wells, the better the test results will be.

## 7 Validation and characterization*.*

To validate the operation of the ASMI and its ability to determine reliable measurements, tests were performed on PDMS which has a known elastic modulus between 1.3 and 3.0 MPa [26]. PDMS was prepared by mixing 10:1 base:crosslinker of Sylgard 184 curing it at 65 °C for 30 min in an oven. This sample was chosen due to its well-studied properties and high accessibility, making it very practical to use as a baseline to validate the system. This resulted in the data shown in Table 2. These results support the accuracy and precision of the ASMI.

**Table 2:** Validation data on PDMS with a known elastic modulus between 1.3 and 3.0 MPa [26].

Sample #	1	2	3	4	5	6	Average Elastic Modulus (MPa)	Expected Elastic Modulus (MPa)
Measurement	1.7	1.7	1.7	1.7	1.6	1.7	1.70 ± 0.04	1.3–3.0

Characterization of Hardware Performance:

• Average sample acquisition time: 5 min
• Number of samples per test batch: 96
• Sample Size: 100–370 µL
• Range of best suited elastic moduli: 7 kPa − 67 MPa

**Fig. 17:**
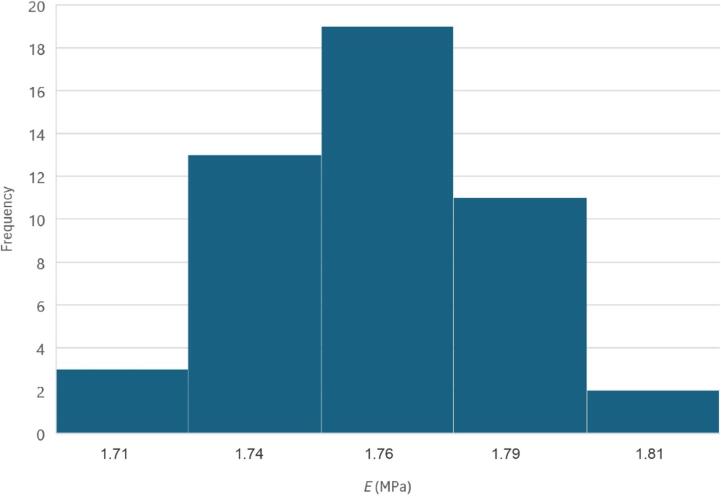
Histogram of 48 measurements of *E* on a PDMS sample taken hourly over two days.

To test the accuracy and stability of the ASMI, measurements were taken on a PDMS sample every hour for two days for a total of 48 measurements. These measurements resulted in a standard deviation of 23 kPa, or 1.3 % of the average measured elastic modulus of 1.76 MPa. This demonstrates that the stability of the force sensor is not a significant concern despite it being an educational system. A histogram of these measurements is show in Fig. 17.

In order to test the generality of these results for stiffer materials and those not in 96-well plates, we performed a series of tests using commercially available thermoplastic polyurethanes. In particular, using comparatively stiff (Ninjatek – Cheetah) and soft (Ninjatek – Ninjaflex) materials, rectangular sample coupons were printed using fused filament fabrication that were 3 × 3 cm^2^ in cross section and 2 cm tall. Samples were printed with a nozzle temperature of 260 °C using a Bambu Lab A1 printer. In addition to being tested using the ASMI, these samples were also tested under compression using a universal testing machine (UTM – Instron 5965) with a 5 kN load cell. To approximate the light indentation of the ASMI, a modulus was extracted from the UTM by fitting the stress vs. strain curve to a line in the range 1 to 2.5 % strain. Collectively, the ASMI was within 30 % of the UTM-measured values in both cases (Table 3), confirming that the ASMI provides valuable results for bulk samples not in well plates.

**Table 3:** Validation of ASMI results collected on 3D printed NinjaFlex and Cheetah thermoplastic polyurethane samples along with reference elastic moduli obtained using bulk compression testing.

Measurement	1	2	3	4	5	6	Average Elastic Modulus (MPa)	Reference Elastic Modulus (MPa)
Ninjatek NinjaFlex® Measurements	1.4	1.6	1.4	1.6	1.6	1.3	1.5 ± 0.1	2.3 ± 0.2
Ninjatek Cheetah™ Measurements	16	17	16	18	18	18	17 ± 1	27 ± 9

In addition to these samples, tests were performed on polyacrylamide-based hydrogels synthesized in a multiwell plate (unpublished method) for *in vitro* cell mechanobiology studies. The polyacrylamide hydrogels were polymerized according to previously published ratios of acrylamide:bis-acrylamide in order to have an estimated elastic moduli ranging from 5 to 135 kPa [27].The expected values in Table 4 were previously validated via nanoindentation measurements on polyacrylamide gels polymerized on coverslips [28]. The ASMI was used to test these materials autonomously over the course of a couple hours, resulting in the following measurements tabulated in Table 4, taken on six sets of six hydrogels. There is known to be some variability due to both pipetting and inconsistent reaction conditions such as temperature and UV exposure, though this uncertainty is difficult to quantify [29].

**Table 4:** Validation data from samples with estimated elastic moduli between 5 kPa and 135 kPa.

Well Row/ Column	1	2	6	7	11	12	Average Elastic Modulus (kPa)	Expected Elastic Modulus (kPa)
A	6.7	7.7	3.7	4.7	3.5	10	6.0 ± 2.5	5.0
B	10	19	11	8.5	9.0	9.9	11 ± 3.9	10
C	8.3	27	22	23	21	16	19 ± 6.5	15
D	26	43	15	34	51	43	35 ± 13	25
E	75	64	48	70	78	73	68 ± 11	70
F	410	190	140	200	170	110	200 ± 100	135

Considering the ASMI as a tool for high throughput experimentation, the system has several advantages in terms of sample consistency. First, being automated, it is possible to take more data than would be practical with an manual system. Additionally, the system collects additional data during the experimentation process that can be used to assess sample consistency. In particular, the system measures the height of the sample, which can be used as a diagnostic measurement to assess sample repeatability and time-variable effects such as dehydration. Finally, the output of this system provides rich opportunities for subsequent analysis. While there is no outlier analysis built into this existing software, the ASMI makes it possible to track all the relevant metadata and increase the experimental throughput to the point where sufficient data exists to maintain good statistics even after manually discarding outliers if needed.

## CRediT authorship contribution statement

**Dylan List:** Validation, Software, Investigation. **Alan Gardner:** Software. **Isabella Claure:** Validation, Resources. **Joyce Y. Wong:** Validation, Resources, Conceptualization. **Keith A. Brown:** Supervision, Resources, Project administration, Conceptualization.

## Declaration of competing interest

The authors declare that they have no known competing financial interests or personal relationships that could have appeared to influence the work reported in this paper.
